# Brown Macroalgae (Phaeophyceae): A Valuable Reservoir of Antimicrobial Compounds on Northern Coast of Spain

**DOI:** 10.3390/md20120775

**Published:** 2022-12-12

**Authors:** Susana Rubiño, César Peteiro, Teresa Aymerich, Maria Hortós

**Affiliations:** 1 Institute of Agrifood Research and Technology (IRTA), Food Safety and Functionality Program, Finca Camps i Armet s/n, 17121 Girona, Spain; 2 Oceanographic Centre of Santander (COST-IEO), Spanish Institute of Oceanography of the Spanish, National Research Council (IEO, CSIC), Marine Culture Units “El Bocal”, Seaweeds Unit, Barrio Corbanera s/n., 39012 Santander, Spain

**Keywords:** brown macroalgae, antimicrobial activity, polyphenols, carbohydrates, proteins, volatile organic compounds (VOCs)

## Abstract

The search for new sources of antimicrobial compounds has become an urgent need, due to the threat that the spread of bacterial resistance represents for global health and food safety. Brown macroalgae have been proposed as a great reservoir in the search for novel antimicrobial compounds. In this study, mid-polarity extracts were performed with a selection of 20 brown macroalgae species from northern Spain. The total polyphenol, carbohydrate and protein contents were quantified by spectrophotometry. The volatile organic compounds (VOCs) of whole macroalgae were also studied as a biomarker of their metabolic state in the representative species of the tested families by gas chromatography-mass spectrometry (GC-MS). The antimicrobial potential of the extracts was assessed by a disk diffusion assay against 20 target bacteria and further determinations of the minimum inhibitory (MIC) and minimum bactericidal concentrations (MBC) were performed by a microdilution assay for the active extracts. *Ericaria selaginoides*, *Bifurcaria bifurcata* and *Dictyota dichotoma* showed an antimicrobial effect against six Gram-positive strains: *Bacillus cereus*, *Bacillus subtilis*, *Geobacillus stearothermophilus*, *Listeria monocytogenes*, *Staphylococcus aureus* and *Staphylococcus haemolyticus*. The phenolic content was generally higher in the extracts that showed antimicrobial activity, followed by carbohydrates and low contents of proteins. The results obtained in this study reveal the potential of brown macroalgae as a promising alternative source of antimicrobial compounds as functional ingredients for the application in industrial fields.

## 1. Introduction

Marine ecosystems harbor a high variety of organisms with a unique composition. Among them, macroalgae (commonly referred to as seaweeds) are important primary producers in oceanic aquatic food webs, consequently contributing to marine ecosystems [1]. In the intertidal zone, macroalgae are arranged in a vertical gradient of diverse communities subjected to either fluctuations or a gradual shift by the incidence of environmental and biological factors. To cope with these harsh and competitive marine conditions, seaweeds have developed an adaptive mechanism against biotic and abiotic stressors based on a set of physiological responses that results in the production of a wide diversity of compounds different than those observed in terrestrial environments [2].

Such chemical variety constitutes a great reservoir for the discovery of novel bioactive compounds. In fact, more than 15,000 primary and secondary metabolites from macroalgae of different chemical natures, including proteins and peptides, polysaccharides, polyphenols, polyunsaturated fatty acids or pigments, have been reported with antioxidant, antimicrobial, antifungal, anticancer or anti-inflammatory activities [3,4,5].

The search for sustainable sources and demands for compounds of a natural origin are in the spotlight, since the potential harmful side effects for health, due to the use of synthetic substances, are under discussion [6,7]. In this regard, macroalgae biomass represent a renewable, varied and versatile source that can be harvested directly in the sea in an environmentally safe way, avoiding the competitiveness for the use of land and the overexploitation of other natural resources [8]. Simultaneously, their production from aquaculture also becomes a benefit for the environment, since these aquatic organisms have the ability to assimilate carbon dioxide from anthropogenic emissions, in addition to nitrogen sequestration, contributing to mitigating climate change [9,10,11].

In addition to the use of macroalgae as a whole food, their singular composition makes them a useful resource of natural ingredients suitable for several industrial applications. In Europe, interest in seaweeds has increased exponentially over the last years and, although their exploitation is still limited, several initiatives are currently ongoing. Seaweeds used as food and food supplements and classified as novel are subject to the pre-market authorization requirements of the novel food regulation (EU) 2015/2283 [12] before they can be freely placed in the European market without the need for pre-market novel food authorization [13].

The need to intensify the search for novel food antimicrobials is increasing since food products are perishable by nature and foodborne contamination is still an important public health issue. Growing consumer demands for high-quality products, coupled with less use of synthetic food additives and minimally processed products with a longer shelf life, promote the development of alternative solutions for food safety to continue playing a key role in the food industry. Moreover, currently, there is also a social need for new antimicrobial compounds since their inadequacy and overuse have increased the spread of antimicrobial resistance, leading to the re-emergence of bacterial diseases, and causing an important clinical, economic and social impact [14,15].

The potential of marine macroalgae as a source of antimicrobials has been studied among the three main phyla. However, interest in brown macroalgae has been extensively described because of their particular composition in polyphenols such as phlorotannins; polysaccharides such as fucoidans; and pigments such as fucoxanthin, that have been widely reported to be active against pathogenic and spoilage bacteria [16], though the antimicrobial activity from crude extracts is not usually attributed to a single compound but could be related to a combination of metabolites.

In this sense, research efforts are needed to explore the richness and potential of local biodiversity in order to fulfill biomass demands and contribute to improving the overall quality and traceability of the seaweed products industry [17]. In this sense, the north of Spain coastline has been divided into four ecological zones according to their environmental differences, which makes this region a good reservoir with great macroalgal diversity [18]. Consequently, a good selection of the species is essential for further steps, and native species well-adapted to the local environment seem to be the best suited [19].

The aim of this study was to assess the antimicrobial activity of brown macroalgae mid-polarity extracts from collected macroalgal species from the northern coast of Spain, contributing with this research the knowledge of its potential as a sustainable and affordable source of novel biocompounds. This work was focused mainly on their potential against foodborne pathogens and spoilage bacteria and their use as natural preservative ingredients, although their application could be extended to other fields such as the pharmaceutical or cosmetic industries.

## 2. Results and Discussion

### 2.1. Volatile Compounds in the Biomass of Seaweeds

**Figure 1:**
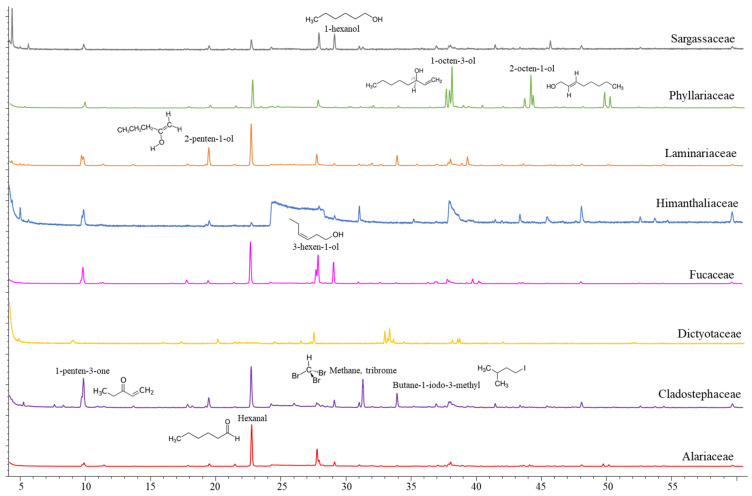
Representative chromatograms of the volatile organic compound profile of whole macroalgae samples from each family.

**Figure 2:**
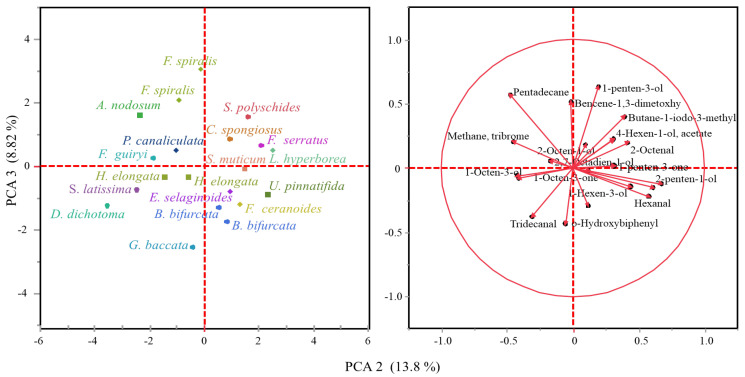
Principal component analysis on volatile compounds data.

Though scarce research has been carried out on volatile organic compounds from macroalgae, at least 200 different volatile compounds have been reported in important commercially brown (*Himanthalia elongata*, *Laminaria* spp., *Laminaria ochroleuca* and *Undaria pinnatifida*) and red macroalgae (*Porphyra umbilicalis* and *Palmaria palmata*) [20]. The volatile organic compounds tentatively identified in whole brown macroalgae collected from the northern coast of Spain are listed in Table 1 and representative chromatograms of the evaluated families are shown in Figure 1. The VOC profile from the mid-polarity extracts was also evaluated (data not shown), although a scarce number of compounds were released under established conditions. Compound classes observed in whole algae samples are in accordance with those described in the literature, since ketones, alcohols, aldehydes, hydrocarbons, esters and halogenated compounds have been identified, with alcohols and aldehydes being the most predominant chemical structures [20]. In this study, differences in the volatile organic compound profile have been observed among taxonomical families (Figure 1), though the principal components analysis (PCA) explains only 22.62% of the variability among the samples (Figure 2). The C5 and C6 volatiles were the most common among the identified compounds, with hexanal and 1-hexanol being identified in a greater number of species, whereas compounds such as o-hydroxybiphenil (aromatic compound) have also been identified in a minor presence. 

Hexanal and the pentyl leaf (C5) volatiles were detected among the evaluated species, with 1-penten-3-one being prominent in *H. elongata* and *Cladostephus spongiosus* and 3-penten-2-ona-4-methyl in the Sargassaceae family. The remaining C6 volatiles had a higher contribution to the volatile organic compound profile of the Sargassaceae species in comparison to the Fucaceae species, whereas their occurrence differs among the Laminariales species. 2-hexene-3,5,5-trimethyl was prominent in the profile of *Saccharina latissima*, 2-hexenal in *Laminaria hyperborea* and *U. pinnatifida*, though the major volatile compound detected in *U. pinnatifida* was 1-octen-3-ol. Significant emission of the C8 volatiles was also seen in *Dictyota dichotoma*, and both the C8 and C9 volatiles were identified in the emission profile of *Sacchoriza polyschides*. The saturated hydrocarbon chain length of the C15 was mostly present in the Fucaceae species, being the major volatile compound detected in *Ascophyllum nodosum*, *Fucus guiryi* and *Pelvetia canaliculata* and showing a remarkable content in *Fucus serratus*. Both the C15 and C13 saturated hydrocarbons were also observed in *D. dichotoma*. Volatile compounds related to organoleptic attributes, such as odor or taste, are currently of interest regarding their potential as ingredients in industrial applications. Short-chain aldehydes and their derivates are known to be important flavor compounds in several foods, and the observed differences in the volatile compound profile should affect the consumer’s acceptability of seaweed-containing food products. C6 and C9 aldehydes, such as n-hexanal and 2-nonenal detected here, have been described as contributors of flavor in *Saccharina angustata* (as *Laminaria angustata*) [21]. Thus, the six-carbon (C6), nine-carbon (C9) and pentyl leaf (C5) volatiles are responsible for the green, earth and herb notes, whereas the occurrence of the eight-carbon (C8) compounds, even at low levels, are responsible for the mushroom, musty or earthy notes of several foods.

Many of the detected volatiles are derived from the degradation of PUFAs through the lipoxygenase pathway (LOX) such as n-hexanal and a set of short-chain unsaturated aldehydes, their alcohols and esters [22,23]. Oxylipin-derived volatiles are quickly released in response to the biotic and abiotic stressors or in tissue injury due to mechanical wounding [24], but relatively low levels of these volatiles have been emitted in intact and healthy plants. Thus, the minor number of volatile organic compounds observed in this study could be attributed to the careful conditions applied in the sample collection and transport, as well as the use of cryogenic disruption of tissues and the mild experimental conditions applied in the volatile analysis from the frozen samples.

Furthermore, hexanal, together with other C6 compounds such as 2-hexenal, has been reported to show an antimicrobial effect against foodborne pathogenic microorganisms such as *Escherichia coli*, *Salmonella enterica* and *Listeria monocytogenes* [25], showing a great potential as a food preservative. However, these compounds have not been identified in the evaluated species of macroalgae that showed antimicrobial activity.

Halogenated compounds, brominated and iodinated species have also been identified, tribrome-methane or bromoform was observed in *A. nodosum*, *D. dichotoma*, butane-1-iodo-3-methyl in *L. hyperborea*, and both compounds were identified in *C. spongiosus*. Halogenated compounds are influenced by nutrient concentration, temperature and salinity conditions, although the release of volatile compounds is mostly affected by light variations [26]. In addition, the occurrence of halogenated volatiles has also been proposed as a biomarker of the macroalgae physiological state since they involve defense mechanisms against grazing and as a response to light exposure. Specifically, Ohsawa et al. (2001) [27] described bromoform as an agent to eliminate epiphytic organisms in the red macroalgae *Corallina pilulifera*. Although not related to their antimicrobial potential, it is interesting to mention that bromoform has also been identified in species of the genus *Asparagopsis* (*A. taxiformis* and *A. armata*), which have recently been postulated as effective feed ingredients in the dietary intervention of ruminants for methane mitigation. The feed inclusion of these species at less than 1% of the feed organic matter reduces methane emissions from sheep and cattle [28,29].

In the mid-polarity extracts, although a minor number of compounds have been released (data not shown), it is of interest to highlight that chlorinated and iodinated compounds, such as 2-propanone-1-chloro and 1-iodo-2,3-epoxypropane, have only been detected in species belonging to the Laminariaceae, *L. hyperborea* and *S. latissima* families. This finding agrees with the description of Laminariales as the major accumulators of iodine within living organisms and as a great source of iodocarbons in coastal environments [30].

### 2.2. Chemical Composition of Extracts

The current need for novel antimicrobial compounds has placed macroalgae in the spotlight because of their richness in bioactive compounds. Specifically, brown macroalgae are of great interest as they harbor compounds that are exclusively found within this class [31]. Among them, interest has been focused on molecules such as phlorotannins, phenolic compounds; fucoidans, sulphated polysaccharides; and fucoxanthin, carotenoid pigment, because of their numerous properties, including their antimicrobial potential [32,33,34]. Other compounds that are not exclusively from Phaeophyceae, such as proteins, peptides and fatty acids, have also been described to show antimicrobial properties [16].

Over the last decades, different extraction techniques and solvents have been used for the extraction of these compounds, with solid–liquid extraction (SLE) traditionally being performed [35]. The extraction of chemical structures depends on a wide range of factors such as the polarity of solvents, temperature, time and the extraction technique being performed. Quantification methods, as well as the availability of standards, also influence the evaluation of the obtained results since the specific identification and quantification of metabolites are not always likely to be performed due to the lack of suitable standards from the marine environment. Moreover, it is well known that numerous natural factors strongly influence macroalgae composition: biotic factors, including intrinsic (i.e., growth, reproduction state) and extrinsic factors (i.e., herbivory, epiphytism); and abiotic factors (i.e., latitude, season, irradiance, depth) [36]. This current context and the non-existence of standardized protocols originate a high variability and make it hard to establish proper comparisons with other results found in the literature. In this work, the spectrophotometric detection of the phenolic, carbohydrate and protein content of the mid-polarity extracts (Table 2) has been evaluated in selected brown macroalgae.

In general, the total phenolic content was higher in most evaluated species (29.28–115.13 mg PGE/g dw), whereas *S. latissima* (33.43 mg GE/g dw) and some species from the order Fucales exhibited a higher carbohydrate content (15.14–19.15 mg GE/g dw). The protein content was generally minor in all samples (Table 3).

The values for these compounds in the present study varied between species and families as a result of the factors previously described. The most specific polyphenols of brown algae, phlorotannins, are subjected to inter-species variations [37]. The higher phenolic content has been observed in species belonging to the Fucales and within the Sargassaceae, Fucaceae and Himanthaliaceae families, whereas the contents in the Alariaceae, Laminariaceae, Cladostephaceae and Phyllariaceae families were, in general, the lowest ones, ranging between 2.52 mg PGE/g dw in *S. latissima* and 6.25 mg PGE/g dw in *U. pinnatifida*.

In the order Fucales, phlorotannins may amount to up to 20% dw [38]. In this study, the highest value was observed for *Halidrys siliquosa* (115.13 mg PGE/g dw), followed by *Fucus vesiculosus*, *F. serratus* and *A. nodosum* (42.64–54.58 mg PGE/g dw). There were also species with a low phenolic content among members of the Fucaceae family, such as *Fucus ceranoides* (8.44 mg PGE/g dw) and some species of *F. guiryi* and *P. canaliculata*. Whereas the intermediate values ranged between 19.28 and 34.63 mg PGE/g dw for species from the Sargassaceae and Dictyotaceae families, as well as some Fucaceae.

These results are in accordance with the values reviewed by Jacobsen et al. (2019) [39], who indicate the highest concentrations for large species of brown macroalgae, such as *Fucus* sp., *Sargassum* sp. and *A. nodosum*; although, in this work, *Sargassum muticum* had the lowest value quantified among the species of the Sargassaceae family with 13.31 mg PGE/g dw. A high variability in the phenolic content has been reported for *A. nodosum*. Tierney et al. (2013) [40] reported values above 100 mg PGE/g in extracts performed from a fresh sample with water and organic solvents, whereas low values can also be found, ranging between 0.11 and 0.17 mg PGE/g dw in water and acidic extracts performed by solid–liquid and ultrasound extraction methods [41]. Since there are not standardized procedures for the extraction of natural antimicrobials, the reviewed data highlight the influence of different techniques and extraction solvents on their recovery as well as their antimicrobial potential. Special attention should be paid concerning the use of high temperatures during processing. Thus, despite it helping to obtain higher yields, its application is still under discussion because of the possible thermal degradation of compounds, the hydrolysis of complex molecules in smaller structures or even polymerization reactions [42]. In methanolic extracts (60%), the reported extraction yields obtained at 60 °C were around ten times lower [43] than the recorded values at 40 °C [44], although the influence of other factors cannot be disregarded. Moreover, low yields in extracts performed with hot water have also been described [45]. In this work, extractions have been performed at room temperature to avoid a thermal effect over the molecular structures and the possible bioactivities of the extracted compounds. Thus, considering the scaling of future extraction methods at an industrial level, decreasing the energy demands would make these processes more affordable and sustainable.

Regarding the carbohydrate content, the higher values were observed in the Laminariaceae, Fucaceae and Sargassaceae families, with the highest contents being for *S. latissima* (33.43 mg GE/g dw), *F. ceranoides* (19.15 mg GE/g dw) and *H. siliquosa* (18.95 mg GE/g dw), followed by *F. serratus* and *A. nodosum* (15.61 and 15.14 mg GE/g dw, respectively). The results for *H. siliquosa* agree with the values obtained by Olsson et al. (2020) [46], that also reported the highest carbohydrate content for this species collected on the Swedish west coast. *S. latissima*, commonly known as sugar kelp, has a great commercial value as a source of carbohydrates used as emulsifiers and thickeners, because of their high content (30 to 50% dw), especially in alginate, which represents up to 40% dw [47]. The intermediate levels ranged between 6.05 and 13.57 mg GE/g dw, including species from all the families, and minor values (≤4.65 mg GE/g dw) were registered for *S. polyschides*.

In general, the results for the carbohydrate content reported in the bibliography are higher than those obtained in this work. Higher values, that amount to up to 100 times or more, have been described in ethanolic extracts prepared from commercial macroalgae such as *S. latissima*, *U. pinnatifida*, and *H. elongata*, collected on the Atlantic coast of Galicia. The values were also higher in other Laminariaceae species such as *L. ochroleuca* [48]. This fact could be mainly attributed to the extraction medium used. Due to their high polarity, the extraction of polysaccharides has been currently performed with hot water or acidic solutions, although other studies employed polar solvents such as ethanol [49]. Nevertheless, a mid-polarity extraction medium was selected in this work in order to minimize the solubilization of the major carbohydrates that occurs in brown macroalgae since their extraction could make further clean-up steps difficult. Moreover, since the extraction procedures influence the composition of the extracts [50], the lower content achieved in this work could also be a result of the selection of carbohydrates with specific chemical or functional structures.

Phaeophyceae has the lowest protein content among the three macroalgae phyla, representing 3–15% dw [51]. Proteinic structures were the minor component in these extracts. The higher values were observed among members of the Fucaceae and Himanthaliaceae families and for *H. siliquosa* in Sargassaceae, with amounts from 5.33 to 10.74 mg BSAE/g dw. However, a high variability is also observed for the proteinic content and there are exceptions in these families with low values, such as *F. ceranoides* and *S. muticum* (≤2.78 mg BSAE/g dw). The rest of the species from Sargassaceae also registered minor concentrations (≤2.78 mg BSAE/g dw), except for *Gongolaria baccata* (3.57 mg BSAE/g dw) and fertile specimens of *Bifurcaria bifurcata* (3.67 mg BSAE/g dw). Most of the studies found in the literature determine the total protein content in the macroalgae, which makes it difficult to establish comparisons. However, the values obtained in this study were below the percentages described above for brown macroalgae (3–15% dw), with the highest ones being in some specimens of *P. canaliculata* and *H. elongata*, with just 1% of dry weight protein content.

In addition, it is worth highlighting the differences observed regarding the life stage in *B. bifurcata*. No significant differences were observed in the carbohydrate and protein content between the vegetative and reproductive samples; however, the phenolic content was significantly different in the reproductive samples (*p* ≤ 0.01). A higher phenolic content during the reproductive stage could be related to their defense function to protect themselves, especially during the reproductive stage [52], although this effect was not observed in *H. elongata* since the phenolic content was higher (*p* ≤ 0.01) in the vegetative specimens and other factors, such as season (spring and summer), could have an influence. Similarly, the non-fertile specimens of *P. canaliculata* collected in Galicia in 2019 showed a lower proteinic content (6.36 ± 0.67 mg BSAE/g dw) and higher total phenolic (14.06 ± 0.31 mg PGE/g dw) content than specimens collected in Cantabria in 2017 (10.03 ± 0.67 mg BSAE/g dw and 3.17 ± 0.31 mg PGE/g dw, respectively).

Moreover, some species were collected under the different conditions of season, year or location, which affected the composition of the mid-polarity extracts. Carbohydrates and the total phenolic content were higher (*p* ≤ 0.01) in specimens of *F. guiryi* collected in 2019 (7.71 ± 0.28 mg GE/g dw and 29.33 ± 1.46 mg PGE/g dw, respectively), with respect to those collected in 2017 (≤4.65 mg GE/g dw and 2.45 ± 1.46 mg PGE/g dw, respectively). Similarly, the extractability of polyphenolic (*p* ≤ 0.01) and proteinic compounds (*p* ≤ 0.01) was higher in specimens of *H. elongata* collected in spring (48.31 ± 1.25 mg PGE/g dw and 10.74 ± 0.22 mg BSAE/g dw, respectively) than those collected in summer (25.47 ± 1.25 mg PGE/g dw and 8.40 ± 0.22 mg BSAE/g dw, respectively). The geographic location affected the concentrations of the total phenolic compounds (*p* ≤ 0.01) and carbohydrates (*p* ≤ 0.01) of the mid-polarity extracts of *Fucus spiralis*. The specimens collected in Comillas exhibited a higher content in the extractable phenolic compounds (24.89 ± 0.82 mg PGE/g dw), while the extracted carbohydrates (10.68 ± 0.27 mg GE/g dw) were higher in specimens collected in A Coruña. Differences considering these factors have been evaluated by other authors in valuable macroalgae [38,53,54], although, in this sense, more efforts are needed to determine the influence that each factor exerts over the content of certain macroalgae compounds.

### 2.3. Antimicrobial Activity of Macroalgae Extracts

The antimicrobial activity of the mid-polarity extracts from brown macroalgae collected in this study was observed in three species, *B. bifurcata* and *Ericaria selaginoides* from the Sargassaceae family, and *D. dichotoma* from the Dictyotaceae family (Table 4). Irrespective of the considered species, all the extracts exhibited the same spectrum of antimicrobial activity, which is constituted by six Gram-positive bacteria, including foodborne pathogens such as *L. monocytogenes*, *Bacillus cereus* and *Staphylococcus aureus* [55]; spoilage microorganisms such as *Geobacillus stearothermophilus*, spore-forming bacteria that remain a major challenge in the dairy industry [56]; the opportunistic pathogen *Staphylococcus haemolyticus*, and the spore-forming food contaminant *Bacillus subtilis*; thus being of relevant interest for the food industry and human health.

Nevertheless, despite having the same antimicrobial spectrum, the overall quantitative antimicrobial potential profile of each macroalgae extract against the six target strains was different, as determined by the MIC/MBC values obtained for *E. selaginoides* and *B. bifurcata* (Table 5). The scarce occurrence of *D. dichotoma* on the northern coast of Spain did not allow for the estimation of the MIC/MBC values.

The ability of extracts to inhibit Gram-positive strains, not observed against Gram-negative, could be associated with differences in their cell wall structure that determine their permeability, with Gram-negative bacteria being generally more resistant since their outer membrane can act as a barrier to certain compounds [57]. Reported differences in the antimicrobial effect of different macroalgae extracts denote the importance of the choice of solvent in the selectivity of the bioactive compounds. An antimicrobial effect of ethanol and dichloromethane extracts from Atlantic *B. bifurcata* against Gram-positive strains, such as *B. subtilis* and *S. aureus*, has also been described by El Wahidi et al. (2015) [58]. However, Alves et al. (2016) [59] reported antimicrobial activity against Gram-negative strains of dichloromethane extracts from *B. bifurcata*, while methanolic extracts were active against both Gram-positive and Gram-negative bacteria.

*Bacillus* and *Geobacillus* genera are ubiquitous in nature and are of concern in the food industry since they can be present in fresh and pasteurized food due to their ability to generate heat-resistant spores under harsh environmental conditions [60]. *G. stearothermophilus* exhibited a great sensitivity to the *B. bifurcata* and *E. selaginoides* extracts, with the lowest MIC/MBC values and inhibition halos slightly smaller than those obtained for *L. monocytogenes.* The diameter of the inhibition zones observed by a disk diffusion assay was 14.6–18.3 and 13.4 mm, respectively, while the MIC/MBC values ranged between 0.3–0.4 mg/mL for *B. bifurcata* and 0.2–0.3 mg/mL for *E. selaginoides*. This target strain can be found as a contaminant of dairy products and can also be isolated from dried soups and vegetables and, although it is not described as pathogenic, its occurrence in a high concentration reduces the value of the food products [61,62].

Remarkable variations in the antimicrobial activity against *B. cereus* were also observed on both the *B. bifurcata* and *E. selaginoides* extracts. The antimicrobial activity of *B. bifurcata* was higher in the extracts from the vegetative specimens, whereas the highest values observed for the fertile specimens were either for MIC (3.2 mg/mL) or MBC (6.4 mg/mL). The results also differed among the specimens of *B. bifurcata* and *E. selaginoides* collected in 2017 and 2019 and in different locations, ranging between 0.4–6.4 mg/mL and 0.9–4.5 mg/mL, respectively. *B. cereus* is a well-documented food-poisoning microorganism widely distributed in the environment, being responsible for most notified causes of diarrheal and emetic illnesses in developed countries [63], and is found in a wide variety of foods, such as dairy products, meat, pasteurized liquid egg, rice, ready-to-eat vegetables and spices [64].

A scarce antimicrobial effect against *B. subtilis* was observed for the *E. selaginoides* extracts, whereas the *B. bifurcata* extracts also showed a great variability. In general, both species showed MIC/MBC values >14.2 mg/mL, although lower MIC values were observed for *B. bifurcata* collected in August 2017 (0.9 mg/mL) and December 2019 (0.4 mg/mL). *B. subtilis* has been safely used in traditional fermented foods [65]; however, although it is not considered as pathogenic, some strains of this species may occasionally cause food poisoning [66] and it has been associated with the spoilage of UHT and canned milk products, as well as the production of toxins under mesophilic temperatures [67].

*B. cereus* and *B. subtilis* are commonly used as target strains in antimicrobial assays with macroalgae extracts; however, references about the antimicrobial effects of extracts of this nature against *G. stearothermophilus* have not been found in literature. Other authors have reported activity against *B. subtilis* when tested organic extracts performed with specimens of *B. bifurcata* collected in Portuguese and Moroccan regions of the Atlantic Ocean [58,59,68]. Salvador et al. (2007) [69] also reported activity against both *B. cereus* and *B. subtilis*, with solid extracts performed with *E. selaginoides* collected on the Atlantic coast of Spain.

Staphylococci are non-spore-forming bacteria ubiquitously distributed with a high tolerance for salt [70]. The best results for both *S. haemolyticus* and *S. aureus* were obtained from *E. selaginoides* specimens, collected in the same month of different years and locations. In general, lower values were registered for the *B. bifurcata* specimens collected in Galicia in May 2019 and, interestingly, these specimens showed similar results against Staphylococci strains despite their differences in the life stage (vegetative and fertile). *S. haemolyticus* is generally considered non-pathogenic since they are the predominant bacteria of fermented foods worldwide, though their isolation from skin infections in humans and animals places uncertainties around their safety. Nevertheless, *S. aureus* is a well-known virulent pathogen [71], able to produce a wide variety of toxins [72]. The intake of contaminated food, particularly processed meat and dairy products, is generally caused by improper handling and the subsequent storage at elevated temperatures is a major cause of food poisoning.

Unlike *E. selaginoides*, the *B. bifurcata* extracts showed scarce activity against *L. monocytogenes*, foodborne pathogenic bacteria found ubiquitously that can survive under refrigeration and food preservation measures. It can contaminate a wide range of foods, such as raw, chilled and ready-to-eat food (RTE) and, although the incidence of infections is low, it is especially dangerous for pregnant women and vulnerable groups, making this species responsible for many of the foodborne fatalities. Interestingly, the inhibitory capacity of the *E. selaginoides* extracts ranged between 2.3–3.6 mg/mL, being bactericidal at 3.6–4.5 mg/mL. No activity of these macroalgae species against *L. monocytogenes* has been found in the literature, but species belonging to the same genera as *Listeria innocua* were shown to be sensitive to the methanolic extracts of *E. selaginoides* [73].

Antimicrobial activity of the extracts from *D. dichotoma* was also observed by a disk diffusion assay against *B. cereus*, *B. subtilis*, *G. stearothermophilus* and *L. monocytogenes*, with the most sensitive being *G. stearothermophilus*. It also showed a bacteriostatic effect against *S. aureus*. Antimicrobial activity in the extracts of different polarities performed with *D. dichotoma* specimens collected in locations spread worldwide, such as in Spain, India, Serbia and Algeria, also reported activity against *B. cereus*, *B. subtilis*, *S. aureus* or *L. monocytogenes* [69,74,75,76].

Discrepancies between the results performed by disk diffusion and microdilution assays have been observed. In particular, the high antimicrobial activity of *B. bifurcata* extracts against *L. monocytogenes* observed by disk diffusion was not consistent with their high minimum inhibitory concentration. Moreover, the vegetative and reproductive specimens of *B. bifurcata* showed activity against *B. cereus* in the microdilution plate assay but not when they were tested by disk diffusion. Though a specific solution medium was designed to improve the solubility of the mid-polarity crude extracts and their diffusion into the aqueous agar matrix, it has to be taken into consideration that crude extracts are complex mixtures of bioactive compounds with a wide range of polarities, which do not always diffuse as easily as polar compounds. These discrepancies in the results obtained with different methods have been described by many authors who tested the antimicrobial activity of plant extracts, evidencing the effect that methodology may exert over the results, especially when crude extracts are tested [77]. Nevertheless, due to its simplicity and low cost, a disk diffusion assay is usually conducted as a preliminary screening of the antimicrobial activity, whereas a serial microdilution assay provides more accurate information about the inhibitory and bactericidal concentrations.

Furthermore, the variability in the extract composition allows for the consideration that the observed antimicrobial activity is not attributed to a single compound, but to an additive and/or synergic or antagonist action of different compounds. The molecular weight of molecules has also been proved to influence the antimicrobial activity [78]. Thus, the broad spectrum of isolated compounds achieved in the mid-polarity extracts includes pigments such as fucoxanthin [79], sugars and polyphenols, and variations in the content and the composition profile were assumed to be affected by the differences of the genetic lineages and the effect of the environmental factors. In this sense, a seasonal effect on the macroalgae development, as well as the chemical composition of seaweeds, has been reported [54], but the effect on the antimicrobial activity by environmental fluctuations is poorly understood. Thus, a similar antimicrobial effect against *G. stearothermophilus* was detected, irrespective of the season, year and geographical location of collection with extracts performed with the perennial algae *B. bifurcata* in this study. However, according to Čagalj et al. (2022) [80], the antibacterial activity of Mediterranean *Cystoseira compressa* was reported to be higher in summer. On the other hand, the unaffected antimicrobial activity between seasons of *Dictyota indica*, reported by Karkhaneh Yousefi et al. (2020) [81], is probably due to its seasonal development, mainly between spring and summer. In view of these results, no relation between the season and the antimicrobial activity could be established so far. Thus, research in this sense is still in its infancy and more efforts are needed to elucidate the compounds responsible for the antimicrobial effect observed and to determine the main factors involved in the production of these bioactive compounds. Advances in this sense are of great importance in the future in order to establish their production under controlled conditions.

## 3. Materials and Methods

### 3.1. Macroalgae Samples Collection and Processing

Twenty-seven brown algae samples (class Phaeophyceae), including twenty different species, were collected on rocky substrata in the lower intertidal and upper subtidal zones on the Galician (northwest of Spain) and Cantabrian coasts (north of Spain) in different seasons during 2017 and 2019 (Table 6). Taxonomic identification of the specimens was performed based on their morphological characteristics using keys and the specialized literature.

All the individuals of each species were washed with sterile seawater to remove sand and epiphytes and wrapped in sterile cloths moistened with seawater. Then, they were kept under darkness, humid atmosphere (>84%), and cool conditions with ice packs (<15 °C) into expanded polystyrene boxes (EPS) until being transported overnight to IRTA-Monells (Girona, Spain). In order to ensure suitable delivery conditions of the seaweeds, the temperature and relative humidity inside each EPS box were monitored continuously at 10-min intervals using data loggers (Hobo Pendant UA-002-64; Hobo U23 Pro v2, Onset Computer Corporation, Bourne, MA, USA). The temperature and relative humidity during the transportation period in both summer and winter was maintained at a mean temperature of 9.0 ± 2.9 °C, with a maximum of 14.2 °C and a minimum of 5.8 °C, and a mean relative humidity of 91.6 ± 2.6%, with a maximum of 94.8% and a minimum of 84.6%.

Once the samples arrived, they were kept at −80 °C until further processing. In the laboratory, algal biomass was ground to a fine powder using a cryogenic homogenizer (SPEX SamplePrep, Metuchen, NJ, USA) and stored at −80 °C under vacuum and darkness conditions until extracts preparation.

### 3.2. Headspace Solid-Phase Microextraction (HS-SPME) and GC-MS Conditions

The volatile compound profile was performed using solid-phase microextraction (SPME) with a Combi PAL autosampler and separated, identified and quantified in a gas chromatograph 6850 GC-System equipped with a mass selective detector 5975C VL MSD (Agilent Technologies, Santa Clara, CA, USA). Volatile compounds were tentatively identified by comparing their mass spectra with those contained in MassHunter Quantitative Analysis B.05.01 software combined with the National Institute of Standards and Technology database (NIST 2.0 version, Gaithersburg, MD, USA).

The SPME tool was loaded with a stable-flex fiber of divinylbenzene/carboxen/polydimethylsiloxane (DVB/CAR/PDMS), coated with a 50/30 μm thickness (Supelco, Bellefonte, PA, USA). Before the analysis, the fiber was conditioned at 270 °C for 30 min in a SPME Fiber Conditioning Station. For headspace SPME extraction, 1 ± 0.01 g of each sample was weighed in a 10 mL amber vial (Agilent Technologies, Santa Clara, CA, USA), magnetic capped with a PTFE/silicone septum. The extraction was carried out by headspace mode at 40 °C for 40 min with magnetic stirring (250 rpm). The SPME fiber was desorbed in the injection port (splitless mode; helium pressure 34.5 kPa) at 270 °C for 5 min. The capillary column used for volatile compound separation was a DB-5MS capillary column (30 m, 250 μm i.d., 1.0 μm film thickness; J&W Scientific, Folsom, CA, USA). Helium was used as a carrier gas with a constant flow of 0.8 mL/min. The gradient temperature used for the separation was performed at 40 °C (10 min)—4 °C/min (140 °C)—10 °C/min (200 °C)—30 °C/min (250 °C, 5 min). Mass spectra of volatile compounds were acquired over the range *m*/*z* 40–450 in scan acquisition mode, with the mass detector transfer line being maintained at 280 °C, the mass source at 230 °C and the mass quad at 150 °C. Compounds were considered as correctly identified when their spectra presented a library match factor > 95%.

### 3.3. Extraction Procedure

Algae extracts were prepared by triplicate according to Rubiño et al. (2022) [79], using a mid-polarity extraction medium composed of a mixture of hexane–isopropanol–water (10:80:10). Three consecutive extractions of 30 mL each from 3.5 g of a frozen algae sample were performed at room temperature in an orbital homogenizer for 60 min each, and then centrifuged at 5000 rpm for 10 min and at 4 °C. Supernatants were pooled in 50 mL amber flasks. 

Extracts were evaporated to dryness under vacuum conditions at room temperature (Thermo Scientific™ Savant™ SpeedVac™ SPD120 Vacuum, Thermo Fisher Scientific, Waltham, MA, USA). Dry residues were then resuspended under sterile conditions in 1 mL of a medium composed of a mixture of water–glycerol–tween 80 (80:10:10) to solubilize and facilitate the diffusion of the mid-polarity extracts through the agar.

All reagents used were of analytical grade (VWR Chemicals, Radnor, PA, USA and Merck, Darmstadt, Germany) and ultrapure water was obtained by a Milli-Q system (Millipore, Burlington, MA, USA). Because of safety concerns regarding the use of some organic solvents to produce macroalgae extracts with possible further applications, in this study, the current legislation was taken into consideration (2009/32/EC) [83] and food-grade solvents were selected.

### 3.4. Determination of Proximate Composition

#### 3.4.1. Total Phenolic Content

Total phenolic content (TPC) was measured by the Folin–Ciocalteu method adapted to microtiter plate measurement [84]. An aliquot (100 μL) of the extract was placed into a 2 mL conical tub and mixed with 500 μL of the Folin–Ciocalteu reagent, diluted 1:10 (Sigma-Aldrich, San Luis, MO, USA) with water (Milli-Q system, Millipore, Burlington, MA, USA), and 400 μL of 12.5% sodium carbonate (Sigma-Aldrich, San Luis, MO, USA) solution. The mixture was allowed to stand at 45 °C for 15 min. Samples were centrifuged at 5500 rpm for 5 min to remove the excess of sodium carbonate. An amount of 250 μL was transferred into a microtiter plate and the absorbance was read at 765 nm in a Varioskan Flash spectrophotometer (Thermo Fisher Scientific, Waltham, MA, USA). A calibration curve was obtained using phloroglucinol (Sigma-Aldrich, San Luis, MO, USA) as standard and the range of concentrations used was 10–80 ng/μL. Each standard and sample solution were run in duplicate and results were expressed as milligrams of phloroglucinol equivalents (PGE) per gram of seaweed dry weight (mg PGE/g dw). The limits of detection (LOD) and quantification (LOQ) were calculated according to Long & Winefordner (1983) [85], assuming k values of 3 and 10, respectively.

#### 3.4.2. Carbohydrate Content

Total carbohydrate content was evaluated by the phenol–sulphuric acid method [86]. An aliquot (200 μL) of extract was previously dried in a 2 mL conical tube to avoid interferences with the extraction medium during the reaction and then resuspended in the same volume of water. An amount of 200 μL of phenol 5% (Sigma-Aldrich, San Luis, MO, USA) and 1 mL of sulphuric acid 96% (Panreac, Barcelona, Spain) were added and mixed with the residue. Reaction was performed for 15 min at 30 °C. An amount of 250 μL was transferred into a microtiter plate and the absorbance was read at 490 nm in a Varioskan Flash spectrophotometer (Thermo Fisher Scientific, Waltham, MA, USA). Each standard and sample solution were run in duplicate. A calibration curve was obtained using glucose as standard and the range of concentrations used was 25–250 ng/μL. Results were expressed as milligrams of glucose equivalents per gram of seaweed dry weight (mg GE/g dw). The detection (LOD) and quantification (LOQ) limits were determined as described in Section 3.4.1.

#### 3.4.3. Protein Content

Total protein content was determined by Bradford method [87]. An amount of 200 μL of extract was previously dried in a 2 mL conical tube with nitrogen to avoid interferences with the extraction medium during the reaction and resuspended in the same volume of water. An amount of 1 mL of Bradford reactive diluted 1:6 with water (Bio-Rad Protein Assay; Bio Rad Laboratories, Hercules, CA, USA) was added to each sample and the mixture was allowed to stand at room temperature for 30 min. An amount of 250 μL was transferred into a microtiter plate and the absorbance was read at 595 nm in a Varioskan Flash spectrophotometer (Thermo Fisher Scientific, Waltham, MA, USA). Each standard and sample solution were run in duplicate. A calibration curve was obtained using bovine serum albumin (BSA; Sigma-Aldrich, San Luis, MO, USA) as standard and the range of concentrations used was 25–200 ng/μL. The results were expressed as milligrams of BSA equivalents per gram of seaweed dry weight (mg BSAE/g dw). The detection (LOD) and quantification (LOQ) limits were determined as described in Section 3.4.1.

### 3.5. Antimicrobial Activity Screening

#### 3.5.1. Test Microorganisms and Culture Conditions

The antimicrobial activity of the extracts was assessed against 20 target strains, including Gram-positive and Gram-negative strains: [Gram-negative]—*Escherichia coli* LMG (Laboratory of Microbiology Gent) 10266, *Serratia marcescens* ATCC (American Type of Culture Collection) 25419, *Proteus vulgaris* LMG 16708 T, *Pseudomonas aeruginosa* CECT (Spanish Type Culture Collection) 116, *Salmonella enterica* serovar Enteritidis GN91 G5 (CRESA, IRTA), *Salmonella enterica* serovar. Thyphimurium CECT 443 H1, *Citrobacter rodentium* CIP (Pasteur Institute Collection) 104675, *Enterobacter cloacae* LMG 2783 T, *Cronobacter turicensis* LMG 23827 T, *Cronobacter malonaticus* LMG 23826, *Cronobacter sakazaki* LMG 5740 T and *Cronobacter dublinensis* LMG 23825 T; [Gram-positive]—*Listeria monocytogenes* CTC 1011 (Meat Technology Centre, IRTA), *Bacillus cereus* LMG 12335, Bacillus subtilis CECT 4002, *Geobacillus stearothermophilus* CECT 43, *Enterococcus faecalis* CECT 795, *Staphylococcus aureus* CECT 976 and *Staphylococcus haemolyticus* CECT 4900.

All the strains were cultivated from −80 °C stock cultures, cryoprotected with 20% glycerol (*v/v*) for 18 h in TSBYE (Triptic soy broth, VWR Chemicals, Radnor, PA, USA) and supplemented with 0.6% Bacto^TM^ yeast extract (BD Biosciences, Franklin Lakes, NJ, USA). Incubation temperature was 37 °C, except for *Bacillus subtilis* CECT 4002 and *Bacillus cereus* LMG 12335 that were incubated at 30 °C. For optimal growth of *B. subtilis*, shaking (300 rpm) was also required.

#### 3.5.2. Disk Diffusion Assay

Determination of antimicrobial activity of macroalgae extracts was carried out using the overlay and disk diffusion methods. Cultures of each target strains at the early stationary phase (10^7^–10^9^ cfu/mL) were mixed with 20 mL of Müeller Hinton soft agar (0.7%, OXOID CM0337, Thermo Fisher Scientific, Waltham, MA, USA) and poured into sterile plates. The final concentration of bacteria in the overlay was between 10^4^–10^6^ cfu/mL.

Sterile paper disks (6 mm diameter, Fisherbrand^TM^, Thermo Fisher Scientific, Waltham, MA, USA) were disposed over the agar and hydrated with 5 μL of the extract dissolution medium. Then, 20 μL of each extract were applied. The assay was performed in triplicate. One disk with extract dissolution medium was added as a negative control and 1 μL of chloramphenicol (2 mg/mL) was used as a positive control in each plate. Once prepared, plates were incubated at the optimal growth temperature for each strain for 18–24 h.

Antibacterial activity was defined by inhibition zones around the paper disk and their diameters were measured. Extracts that showed antimicrobial activity and target sensitive strains to those extracts were chosen for further determinations.

#### 3.5.3. Determination of MIC (Minimum Inhibitory Concentration) and MBC (Minimum Bactericidal Concentration)

MIC and MBC were evaluated for every active extract using the microdilution method in a 96-well plate according to the criteria recommended by the Clinical and Laboratory Standards Institute with modifications [88]. An amount of 150 μL of 24 h bacterial cultures grown in BHI (GranuCult™ Brain Infusion Heart Broth, Merck, Darmstadt, Germany) at the optimal growth temperature of each strain was added to the wells of the microtiter plate to obtain a final concentration of bacteria in each well of 10^4^ cfu/mL. Row A served as the growth control for each of the assayed strains. In row B, 300 μL plus 20 μL of each extract were added and serial two-fold dilutions were performed to quantify antimicrobial activity. Assays were performed in duplicate for each strain. Plates were incubated at the optimal growth temperature for 18–24 h. The turbidity was visually observed and measured at a wavelength of 600 nm in a Varioskan Flash spectrophotometer (Thermo Fisher Scientific, Waltham, MA, USA). The minimum concentration that inhibited the growth of the bacteria was determined as the MIC. To assess the MBC, concentration that achieved an inactivation of 99.9% of the bacteria, plate counts for each strain were performed for all the wells with inhibited growth.

### 3.6. Statistical Analysis

The statistical analysis was performed using the software JMP^®^ version 16.0.0 (SAS Institute Inc., Cary, NC, USA). All results were shown as means of replicates. Data were expressed as means ± standard deviations. One-way ANOVA was used to assess significant differences between samples, followed by the Tukey’s comparison test to carry out pairwise comparisons between means. Differences were considered significant at *p* ≤ 0.05. Principal component analysis (PCA) was performed to evaluate macroalgae based on VOCs composition.

## 4. Conclusions

The results obtained in this study highlight the antimicrobial potential of brown macroalgal species that are inhabitants of the northern coast of Spain. Remarkable growth inhibitory effects were observed in the *D. dichotoma*, *B. bifurcata* and *E. selaginoides* specimens against the foodborne pathogens *L. monocytogenes*, *S. aureus* and *B. cereus*, spore-forming bacteria *G. stearothermophilus* and *B. subtilis*, and the nosocomial pathogen *S. haemolyticus*. The spectrum of action provides evidence of their potential use as non-synthetic preservatives due to their ability to inhibit strains that represent a serious risk in the food industry and human health. The broad spectrum of extracted compounds achieved in the mid-polarity extracts includes pigments such as fucoxanthin, sugars and polyphenols and large variations in the extractable compounds’ profile were assumed to be due to differences in the genetic lineages and the effect of environmental factors. Higher amounts of extractable phenolic compounds (29.28–115.13 mg/g dw) were observed in most evaluated species, while the content in carbohydrates was higher in *S. latissima* (33.43 mg/g dw) and some species from the Fucales (15.14–19.15 mg/g dw). More efforts are required to develop a sustainable and responsible production, as well as the standardization of factors affecting the macroalgae metabolism. In addition, further purification steps will help to understand the nature of the active compounds, and to explore the potential synergistic/antagonist relationships between the chemicals. Further research is also needed to establish the most suitable methodology to test the antimicrobial activity of macroalgal extracts and to assure reproducibility and consensus among the scientific community.

## Figures and Tables

**Table 1:** Volatile organic compounds identified in whole brown macroalgae samples.

Compound	Rt	Compound Class	Formula	Ala	Cla	Dic	Fuc							Him	Lam		Phy	Sar			
				*Up*	*Cs*	*Dd*	*Pc*	*An*	*Fg*	*Fc*	*Fs*	*Fv*	*Fsp*	*He*	*Sl*	*Lh*	*Sp*	*Bb*	*Gb*	*Sm*	*Es*
1-Penten-3-ol	7.73	Alcohol	C_5_H_10_O								√	√				√				√	√
1-Penten-3-one	7.82	Ketone	C_5_H_8_O	√	√		√	√	√	√		√		√		√	√		√	√	
2-Penten-1-ol	14.06	Alcohol	C_5_H_10_O		√					√		√				√		√	√	√	√
3-Penten-2-one-4-methyl	15.89	Ketone	C_6_H_10_O															√			
Hexanal	16.17	Aldehyde	C_6_H_12_O	√	√	√				√		√				√	√		√	√	√
2-Hexenal	19.43	Aldehyde	C_6_H_10_O	√						√		√				√	√				
1-Hexen-3-ol	19.52	Alcohol	C_6_H_12_O							√	√	√	√						√	√	√
1-Hexanol	20.30	Alcohol	C_6_H_14_O	√	√		√			√		√	√						√	√	√
Methane, tribrome	21.71	Halogenated compound	CHBr_3_		√	√		√													
Butane-1-iodo-3-methyl	23.41	Halogenated compound	C_5_H_11_I		√											√					
2-Hexene-3,5,5-trimethyl	25.80	Hydrocarbon	C_9_H_18_			√									√		√				
1-Octen-3-one	25.96	Ketone	C_8_H_14_O		√	√	√			√						√	√				√
1-Octen-3-ol	26.08	Alcohol	C_8_H_16_O	√		√			√						√	√	√				
4-Hexen-1-ol, acetate	27.21	Ester	C_8_H_14_O									√									
2-Octenal	29.71	Aldehyde	C_8_H_14_O														√				
2-Octen-1-ol	30.01	Alcohol	C_8_H_16_O			√											√				
2,7-Octadien-1-ol	30.12	Alcohol	C_8_H_14_O														√				
2,6-Nonadienal	33.68	Aldehyde	C_9_H_14_O														√				
2-Nonenal	33.95	Aldehyde	C_9_H_16_O														√				
Benzene-1,3-dimethoxy	34.31	Aromatic compound	C_8_H_10_O										√								
Pentadecane	50.50	Hydrocarbon	C_15_H_32_	√		√	√	√	√	√	√	√	√	√	√	√					
Tridecanal	50.79	Aldehyde	C_13_H_26_O	√		√				√											
o-Hydroxybiphenil	51.12	Aromatic compound	C_12_H_10_O																√		

**Ala**, Alariaceae; **Cla**, Cladostephaceae; **Dic**, Dictyotaceae; **Fuc**, Fucaceae; **Him**, Himanthaliaceae; **Lam**, Laminariaceae; **Phy**, Phyllariaceae; **Sar**, Sargassaceae; ***Up***, *Undaria pinnatifida*; ***Cs***, *Cladostephus spongiosus*; ***Dd***, *Dictyota dichotoma*; ***Pc***, *Pelvetia canaliculata*; ***An***, *Ascophyllum nodosum*; ***Fg***, *Fucus guiryi*; ***Fs***, *Fucus serratus*; ***Fv***, *Fucus vesiculosus*; ***Fsp***, *Fucus spiralis*; ***He***, *Himanthalia elongata*; ***Sl***, *Saccharina latissima*; ***Lh***, *Laminaria hyperborea*; ***Sp***, *Sacchoriza polyschides*; ***Bb***, *Bifurcaria bifurcata*; ***Gb***, *Gongolaria baccata*; ***Sm***, *Sargassum muticum*; ***Es***, *Ericaria selaginoides*.

**Table 2:** Calibration, linearity, accuracy and precision for polyphenol, carbohydrate and protein content determination by spectrophotometry.

Standard	Wavelength	Equations	R^2^	Linear Range	LOD	LOQ
Phloroglucinol	765 nm	Abs = 17.768 * μg + 0.203	0.999	1–8 μg	0.14 μg	0.47 μg
Glucose	490 nm	Abs = 43.955 * μg − 2.508	0.997	5–50 μg	2.66 μg	8.86 μg
Bovine serum albumin	595 nm	Abs = 45.398 * μg − 1.430	0.998	5–40 μg	1.59 μg	5.30 μg

**Table 3:** Phenolic, carbohydrate and protein content of mid-polarity extracts.

Order, Family/Species	Month/Year	Phenolics ^1^				Carbohydrate ^1^				Protein ^1^			
		mg/g dw			SD	mg/g dw			SD	mg/g dw			SD
**Dictyotales, Dictyotaceae**													
*Dictyota dichotoma*	September 2019	19.73	^f,g,h^	±	1.93	11.10	^d,e,f^	±	0.75	≤2.78			
**Fucales, Fucaceae**													
*Ascophyllum nodosum*	August 2019	42.64	^b,c^	±	9.04	15.14	^b,c^	±	1.06	5.33	^b,c,d,e^	±	0.63
*Fucus ceranoides*	November 2019	8.44	^g,h,i^	±	0.62	19.15	^c^	±	0.66	≤2.78			
*Fucus guiryi*	August 2017	2.45	^i^	±	0.06	≤4.65				6.79	^a,b,c,d,e^	±	0.55
	September 2019	29.33	^d,e,f^	±	3.58	7.71	^e,f,g,h^	±	0.63	7.14	^a,b,c,d,e^	±	0.89
*Fucus serratus*	November 2019	43.87	^b,c^	±	5.12	15.61	^c,d^	±	2.65	8.52	^a,b,c^	±	0.58
*Fucus spiralis*	November 2019	20.10	^f,g,h^	±	1.61	10.68	^d,e,f,g,h^	±	0.07	5.70	^b,c,d,e,^	±	0.76
	December 2019	24.89	^d,e,f,g^	±	1.19	≤4.65				5.85	^b,c,d,e^	±	0.5
*Fucus vesiculosus*	November 2019	54.58	^b^	±	1.25	13.57	^b,c^	±	0.86	5.93	^b,c,d,e^	±	0.52
*Pelvetia canaliculata*	August 2017	3.17	^i^	±	0.26	6.12	^g,h^	±	0.65	10.03	^a,b^	±	1.48
	August 2019	14.06	^g,h,i^	±	0.70	5.15	^h^	±	0.25	6.36	^a,b,c,d,e^	±	0.69
**Fucales, Himanthaliaceae**													
*Himanthalia elongata*	May 2019	48.31	^b,^	±	2.49	6.69	^f,g,h^	±	1.03	10.74	^a^	±	0.40
	August 2019	25.47	^d,e,f,g^	±	1.78	5.51	^g,h^	±	0.23	8.40	^a,b,c,d^	±	0.37
**Fucales, Sargassaceae**													
*Bifurcaria bifurcata*	August 2017	20.87	^e,f,g,h^	±	1.89	12.91	^d,e^	±	1.70	≤2.78			
	May 2019 (vegetative)	24.01	^d,e,f,g^	±	2.41	9.62	^e,f,g,h^	±	1.42	≤2.78			
	May 2019 (fertile)	33.40	^c,d,e^	±	2.24	10.43	^d,e,f,g,h^	±	1.31	3.67	^d,e^	±	1.10
	December 2019	19.28	^f,g,h^	±	1.60	11.34	^d,e,f,g^	±	2.51	≤2.78			
*Ericaria selaginoides*	August 2017	23.06	^d,e,f,g^	±	1.33	12.16	^d,e,f^	±	1.61	≤2.78			
	August 2019	24.74	^d,e,f,g^	±	3.37	9.21	^e,f,g,h^	±	0.68	≤2.78			
*Gongolaria baccata*	August 2017	34.63	^c,d,^	±	2.60	8.74	^e,f,g,h^	±	1.33	3.57	^e^	±	0.27
*Halidrys siliquosa*	November 2019	115.13	^a^	±	16.36	18.95	^b^	±	2.04	6.60	^a,b,c,d,e^	±	1.59
*Sargassum muticum*	May 2019	13.31	^g,h,i^	±	0.51	6.05	^g,h^	±	0.59	≤2.78			
**Laminariales, Alariaceae**													
*Undaria pinnatifida*	May 2019	6.25	^i^	±	0.19	7.76	^e,f,g,h^	±	0.55	3.76	^c,d,e^	±	5.39
**Laminariales, Laminariaceae**													
*Saccharina latissima*	August 2019	2.52	^i^	±	0.06	33.43	^a^	±	2.02	nd,			
*Laminaria hyperborea*	August 2019	2.28	^i^	±	0.12	11.30	^d,e,f,g^	±	0.33	≤2.78			
**Sphacelariales, Cladostephaceae**													
*Cladostephus spongiosus*	September 2019	5.25	^i^	±	0.40	9.93	^d,e,f,g,h^	±	0.65	nd			
**Tilopteridales, Phyllariaceae**													
*Sacchoriza polyschides*	May 2019	4.60	^i^	±	1.10	≤4.65				nd			

^1^*p* ≤ 0.01. Data are presented as mean ± standard deviation (SD) of 3 determinations. Different letters within each row represent significant differences (*p* ≤ 0.05) in phenolic, carbohydrate or protein content.

**Table 4:** Inhibition zones obtained by disk diffusion assay (mm).

Macroalgae Species	Month/Year Collection	Location Collection	Test Organisms—Inhibition Zone (mm)					
			*Bc*	*Bs*	*Gs*	*Lm*	*Sa*	*Sh*
*Bifurcaria* *bifurcata*	August 2017	Cantabria	13.6 ± 0.5	13.0 ± 0.0	17.6 ± 0.0	18.3 ± 1.0	13.6 ± 0.4	15.3 ± 2.3
	May 2019	Galicia	nd	nd	15.7 ± 0.0	18.4 ± 0.6	13.5 ± 0.4	+/−
	May 2019	Galicia	nd	nd	14.6 ± 0.0	17.1 ± 1.5	+/−	nd
	December 2019	Cantabria	12.7 ± 0.5	11.2 ± 0.8	18.3 ± 0.0	19.2 ± 0.4	+/−	14.5 ± 0.2
*Ericaria* *selaginoides*	August 2017	Cantabria	11.7 ± 0.6	11.5 ± 0.9	13.4 ± 0.0	13.9 ± 1.4	12.8 ± 0.0	nd
	August 2019	Galicia	nd	nd	+/−	nd	+/−	+/−
*Dictyota* *dichotoma*	September 2019	Cantabria	10.4 ± 0.0	12.4 ± 0.2	15.2 ± 0.0	10.4 ± 0.0	+/−	nd

Data are presented as mean ± standard deviation (SD) of 3 determinations. ***Bc***: *Bacillus cereus*; ***Bs***: *Bacillus subtilis*; ***Gs***: *Geobacillus stearothermophilus*; ***Lm***: *Listeria monocytogenes*; ***Sa***: *Staphylococcus aureus*; ***Sh***: *Staphylococcus haemolyticus*; nd: non-detected, +/−: bacteriostatic effect.

**Table 5:** MIC and MBC values assessed in microdilution plate assay (mg/mL).

Macroalgae Species	Month/Year Collection	Location Collection	Test Organisms—MIC/MBC (mg/mL)					
			*Bc*	*Bs*	*Gs*	*Lm*	*Sa*	*Sh*
*Bifurcaria* *bifurcata*	August 2017	Cantabria	1.2/1.2	0.9/>19.9	0.3/0.3	>19.9/>19.9	19.9/>19.2	6.5/>13.1
	May 2019	Galicia	0.4/0.4	>24.5/>24.5	0.4/0.4	>24.5/>24.5	12.2/12.2	6.1/6.1
	May 2019	Galicia	3.2/6.4	>25.6/>25.6	0.4/0.4	25.6/>25.6	12.8/12.8	6.4/6.4
	December 2019	Cantabria	0.8/0.8	0.4/>23.9	0.3/0.3	>23.9/>23.9	23.9/23.9	12.0/23.9
*Ericaria* *selaginoides*	August 2017	Cantabria	4.5/4.5	>18.1/>18.1	0.3/0.3	2.3/4.5	2.3/2.3	2.3/4.5
	August 2019	Galicia	0.9/0.9	14.2/>14.2	0.2/0.2	3.6/3.6	3.6/3.6	7.1/7.1

Data are presented as mean of 2 determinations. ***Bc***: *Bacillus cereus*; ***Bs***: *Bacillus subtilis*; ***Gs***: *Geobacillus stearothermophilus*; ***Lm***: *Listeria monocytogenes*; ***Sa***: *Staphylococcus aureus*; ***Sh***: *Staphylococcus haemolyticus*.

**Table 6:** List of collected macroalgae and collection data. The taxonomic classification and currently accepted scientific names of the species, including important synonyms (=), are based on Algaebase and the rules of the International Code of Nomenclature for algae, fungi, and plants (ICN) [82].

Species	Life Stage	Month/Year	Region	Locality	Latitude Longitude	Littoral Zone
**Dictyotales, Dictyotaceae**						
*Dictyota dichotoma* (Hudson) J. V. Lamouroux 1809	Fertile and non-fertile	September 2019	Cantabria	Comillas	43°23′ N 4°17′ W	Subtidal (−1 m)
**Fucales, Fucaceae**						
*Ascophyllum nodosum* (Linnaeus) Le Jolis 1863	Fertile and non-fertile	August 2019	Galicia	As Xubias, A Coruña	43°20′ N 8°23′ W	Mid intertidal
*Fucus ceranoides* Linnaeus 1753	Fertile and non-fertile	November 2019	Galicia	O Burgo, Culleredo	43°20′ N 8°21′ W	Mid intertidal
*Fucus guiryi* G. I. Zardi, K. R. Nicastro, E. S. Serrão & G. A. Pearson 2011 (*=Fucus spiralis* var. *platycarpus* (Thuret) Batters 1902)	Fertile and non-fertile	August 2017	Cantabria	Comillas	43°23′ N 4°17′ W	Upper intertidal
		September 2019	Cantabria	Comillas		
*Fucus serratus* Linnaeus 1753	Fertile and non-fertile	November 2019	Galicia	Esteiro, Muros	42°47′ N 8°58′ W	Low intertidal
*Fucus spiralis* Linnaeus 1753	Fertile and non-fertile	November 2019	Galicia	As Xubias, A Coruña	43°20′ N 8°23′ W	Upper intertidal
		December 2019	Cantabria	Comillas	43°23′ N 4°17′ W	
*Fucus vesiculosus* Linnaeus 1753	Fertile and non-fertile	November	Galicia	Esteiro, Muros	42°47′ N 8°58′ W	Mid intertidal
*Pelvetia canaliculata* (Linnaeus) Decaisne & Thuret 1845	Fertile and non-fertile	August 2017	Cantabria	Comillas	43°23′ N 4°17′ W	Upper intertidal
	Non-fertile	August 2019	Galicia	Santa Cristina, Oleiros	43°20′ N 8°22′ W	
**Fucales, Himanthaliaceae**						
*Himanthalia elongata* (Linnaeus) S. F. Gray 1821	Non-fertile	May 2019	Galicia	Barizo, Malpica	43°19′ N 8°52′ W	Low intertidal and subtidal (−1 m)
	Fertile	August 2019	Galicia	Esteiro, Muros	42°47′ N 8°58′ W	
**Fucales, Sargassaceae**						
*Bifurcaria bifurcata* R. Ross 1958	Non-fertile	August 2017	Cantabria	Trasvia, Comillas	43°23′ N 4°17′ W	Mid intertidal
	Non-fertile	May 2019	Galicia	Portiño, Bens, A Coruña	43°22′ N 8°26′ W	
	Fertile	May 2019	Galicia	Portiño, Bens, A Coruña	43°22′ N 8°26′ W	
	Fertile and non-fertile	Dec 2019	Cantabria	Comillas	43°23′ N 4°17′ W	
*Ericaria selaginoides* (Linnaeus) Molinari & Guiry 2020 [=*Carpodesmia tamariscifolia* (Hudson) Orellana & Sansón 2019] [=*Cystoseira tamariscifolia* (Hudson) Papenfuss 1950]	Non-fertile	August 2017	Cantabria	Trasvia, Comillas	43°23′ N 4°17′ W	Low intertidal and subtidal (−1 m)
	Non-fertile	August 2019	Cantabria	Trasvia, Comillas	43°23′ N 4°17′ W	
*Gongolaria baccata* (S. G. Gmelin) Molinari & Guiry 2020 [=*Treptacantha baccata* (S. G. Gmelin) Orellana & Sansón 2019] [= *Cystoseira baccata* (S. G. Gmelin) P. C. Silva 1952]	Non-fertile	August 2017	Cantabria	Trasvia, Comillas	43°23′ N 4°17′ W	Low intertidal and subtidal (−1 m)
*Halidrys siliquosa* Linnaeus 1753	Fertile and non-fertile	November 2019	Galicia	Santa Cristina, Oleiros	43°20′ N 8°22′ W	Subtidal (−1 m)
*Sargassum muticum* (Yendo) Fensholt 1955	Fertile and non-fertile	May 2019	Galicia	San Pedro de Veigue, Sada	43°20′ N 8°17′ W	Subtidal (−1 m)
**Laminariales, Alariaceae**						
*Undaria pinnatifida* (Harvey) Suringar 1873	Non-fertile	May 2019	Galicia	San Pedro de Veigue, Sada	43°20′ N 8°17′ W	Low intertidal and subtidal (−1 m)
**Laminariales, Laminariaceae**						
*Saccharina latissima* (Linnaeus) C. E. Lane, C. Mayes, Druehl & G. W. Saunders 2006 [= *Laminaria saccharina* (Linnaeus) J. V. Lamouroux 1813]	Non-fertile	August 2019	Galicia	Esteiro, Muros	42°47′ N 8°58′ W	Low intertidal and subtidal (−1 m)
*Laminaria hyperborea* (Gunnerus) Foslie 1885	Non-fertile	August 2019	Galicia	Esteiro, Muros	42°47′ N 8°58′ W	Subtidal (−1 m)
**Sphacelariales, Cladostephaceae**						
*Cladostephus spongiosus* (Hudson) C. Agardh 1817	Non-fertile	September 2019	Cantabria	Comillas	43°23′ N 4°17′ W	Low intertidal
**Tilopteridales, Phyllariaceae**						
*Saccorhiza polyschides* (Lightfoot) Batters 1902	Non-fertile	May 2019	Galicia	Portiño, Bens, A Coruña	43°22′ N 8°26′ W	Low intertidal and subtidal (−1 m)

## Data Availability

Not applicable.
